# What interventions work for suicide prevention? and do they work for the elderly?

**DOI:** 10.1192/j.eurpsy.2021.162

**Published:** 2021-08-13

**Authors:** C. Van Der Feltz-Cornelis

**Affiliations:** Health Sciences, University of York, Heslington, York, United Kingdom

**Keywords:** Suicide prevention, Elderly

## Abstract

**Background:**

Suicides occur more often in the young and in the elderly. However, although several studies have been performed to evaluate the effect of suicide prevention in the young, no studies have explored this in the elderly. Somatic comorbidity is associated with elevated suicide risk, especially in case of pain, which occurs often in the elderly.

**Objective:**

To explore if suicide prevention interventions might be applicable in the elderly and if somatic comorbidity might be relevant for their application.

**Method:**

Evidence synthesis of controlled studies evaluating suicide prevention interventions and of collaborative care trials for depressive disorder in patients with and without somatic comorbidity.

**Results:**

Elderly living alone and with multimorbidity are more prone to suicide risk. Hence interventions involving admission in a general hospital after a suicide attempt, short intervention and follow up might be well applicable in the elderly. In terms of outpatient interventions, and IPD analysis found that collaborative care for depressive disorder is effective in reducing suicidality, especially in the elderly. This effect is independent of somatic comorbidity.

**Conclusion:**

There is potential to develop and evaluate suicide prevention interventions for the elderly. Such interventions should address depression, multimorbidity and social isolation and may be provided at general hospital and at outpatient level.

**Disclosure:**

No significant relationships.

